# Genetic Variations among Different Variants of G1-like Avian Influenza H9N2 Viruses and Their Pathogenicity in Chickens

**DOI:** 10.3390/v14051030

**Published:** 2022-05-11

**Authors:** Amany Adel, Marwa A. Abdelmagid, Ahmed Abd-Elhalem Mohamed, Anishia Wasberg, Zienab Mosaad, Karim Selim, Asmaa Shaaban, Mohamed Tarek, Naglaa M. Hagag, Åke Lundkvist, Patrik Ellström, Mahmoud M. Naguib

**Affiliations:** 1 Reference Laboratory for Veterinary Quality Control on Poultry Production, Animal Health Research Institute, Agriculture Research Center, Giza 12618, Egypt; a.adel18784@gmail.com (A.A.); marwa.abdelmagid@yahoo.com (M.A.A.); vet_t2050@yahoo.com (A.A.-E.M.); drzienabmosaadvet@gmail.com (Z.M.); dr.kareemseleem_87@yahoo.com (K.S.); dr_asmaashaaban@yahoo.com (A.S.); vet.m.tarek@gmail.com (M.T.); naglaahagagahri@gmail.com (N.M.H.); 2 Zoonosis Science Center, Department of Medical Biochemistry and Microbiology, Uppsala University, SE-75121 Uppsala, Sweden; anishia.wasberg@imbim.uu.se (A.W.); ake.lundkvist@imbim.uu.se (Å.L.); 3 Zoonosis Science Center, Department of Medical Sciences, Uppsala University, SE-75185 Uppsala, Sweden; patrik.ellstrom@medsci.uu.se

**Keywords:** avian influenza, pathogenicity, genetic characterization, antigenicity, reassortment

## Abstract

Since it was first discovered, the low pathogenic avian influenza (LPAI) H9N2 subtype has established linages infecting the poultry population globally and has become one of the most prevalent influenza subtypes in domestic poultry. Several different variants and genotypes of LPAI H9N2 viruses have been reported in Egypt, but little is known about their pathogenicity and how they have evolved. In this study, four different Egyptian LPAI H9N2 viruses were genetically and antigenically characterized and compared to representative H9N2 viruses from G1 lineage. Furthermore, the pathogenicity of three genetically distinct Egyptian LPAI H9N2 viruses was assessed by experimental infection in chickens. Whole-genome sequencing revealed that the H9N2 virus of the Egy-2 G1-B lineage (pigeon-like) has become the dominant circulating H9N2 genotype in Egypt since 2016. Considerable variation in virus shedding at day 7 post-infections was detected in infected chickens, but no significant difference in pathogenicity was found between the infected groups. The rapid spread and emergence of new genotypes of the influenza viruses pinpoint the importance of continuous surveillance for the detection of novel reassortant viruses, as well as monitoring the viral evolution.

## 1. Introduction

Influenza A viruses are widely distributed among wild birds, which are considered natural reservoirs for avian influenza viruses (AIVs). Migratory birds can spread different AIV subtypes along their flyways. The low pathogenic avian influenza (LPAI) H9N2 subtype is one of the most commonly circulating LPAI viruses in poultry worldwide [1]. The LPAI H9N2 virus was initially isolated from turkeys in Wisconsin, USA, in 1966 [1]. Since then, this subtype has continued to circulate in poultry populations in several countries, resulting in significant economic loss for the poultry industry [2,3]. In addition to their circulation in birds, sporadic transmission to humans have been reported with LPAI H9N2 viruses in Bangladesh, China, Egypt, and Pakistan [4], posing a threat to public health. To date, LPAI H9N2 viruses have evolved into multiple genotypes and lineages, mainly in Southeast Asia and the Middle East [5]. The LPAI H9N2 viruses of the lineage G1 are the most dominant among domestic poultry and circulate mainly in countries of the Middle East. Influenza A viruses can undergo reassortment when two viruses co-infect the same host cell and exchange their gene segments, leading to the emergence of new subtypes/genotypes [6]. There are several pieces of evidence of natural reassortment involving LPAI H9N2 viruses, and this subtype was involved in the emergence of well-known reassortant viruses, including the highly pathogenic avian influenza (HPAI) H5N1 subtype, the H7N9, and H10N8 subtypes, all known to cause infection in humans [7,8].

In Egypt, LPAI H9N2 was first reported in a quail farm in 2011 (quail/V3413/2011) and spread rapidly to infect several domestic poultry, including chickens, ducks, and turkeys in different locations in Egypt. The virus showed a close phylogenetic relationship to G1-like viruses [9]. Over the past 10 years, LPAI H9N2 viruses have continued to hit Egyptian poultry farms, causing considerable losses for the poultry industry [1,5]. The virus continued to evolve, and a new antigenically distinct variant was found in quail in 2014 (quail/2014 variant) [10]. In the same year, a novel LPAI H9N2 virus variant was detected in pigeons in Egypt as a result of natural reassortment between wild bird-like AIVs (*PB2*, *PB1*, *PA*, *NP*, *NS*) and an Egyptian H9N2/2011 virus (*HA, NA, M*) [11]. Later, in 2014–2015, the pigeon H9N2 virus underwent another natural reassortment event with an Egyptian virus in late 2014, sharing only *PB2*, *PB1*, *PA*, and *NS* [12]. LPAI H9N2 co-infection with other AIVs (e.g., H5N1 and H5N8), as well as other pathogens (e.g., infectious bronchitis virus), was also reported [13]. In late 2017–2018, a novel reassortant, HPAI H5N2 virus emerged with 7 genes of the Egyptian LPAI H9N2 virus and only the *HA* gene from the Egyptian HPAI H5N8 virus [12,14]. These documented examples of reassortment events, including the Egyptian H9N2 LPAI viruses, highlight the importance of continuous genomic and pathogenic monitoring of this virus subtype.

In this study, we aimed to genetically and antigenically characterize different Egyptian LPAI H9N2 genotypes detected in birds in Egypt since 2011 and assess their pathogenesis. Our data emphasize the importance of genetic and pathogenic monitoring of evolving LPAI H9N2 viruses.

## 2. Material and Methods

### 2.1. Viruses and Virus Isolation

Four LPAI H9N2 virus strains were retrieved from the virus bank at the reference laboratory for quality control on poultry production at the Animal Health Research Institute-Egypt (RLQP-AHRI). The virus strains were selected based on the nucleotide sequence of their HA gene segment, as described in our previously targeted surveillance study conducted in 2017–2020 [15]. They were collected during 2017–2018 from vaccinated chicken farms and are hereafter named ch/CAD74/2017, ch/v1172/2018, ch/v1410/2018, and ch/v584/2018 (as detailed in Table S1). Virus propagation and titration were performed via intra-allantoic virus inoculation into specific pathogen-free (SPF) embryonated chicken eggs (ECE) at 9–11 days old embryos, according to the OIE manual [16]. All isolated virus stocks in this study were screened for other viruses, such as HPAI H5, infectious bronchitis virus (IBV), Newcastle disease virus (NDV), and infectious bursal disease virus (IBDV) by real-time RT-PCR [17,18,19].

### 2.2. Genetic and Phylogenetic Analysis

Viral RNA was extracted using the Easypure viral RNA/DNA extraction kit (TransGen Biotech, Beijing, China), according to the instruction manual. The extracted RNA was aliquoted and stored at −20 °C. The RT-qPCR was performed using the extracted RNA within 1–5 days from the extraction time.

Whole-genome amplification of the four selected H9N2 viruses was performed using forward and reverse primers for each gene fragment, as described previously [20]. The fragments were amplified using the Easyscript one-step RT-PCR kit (TransGen, Beijing, China). The amplified products were size separated by agarose gel electrophoresis and purified from the gel using the QIAquick Gel Extraction Kit (Qiagen, Hilden, Germany). The purified products were then used for sequencing reactions using the BigDye terminator kit v3.1 (Applied Biosystems, Waltham, MA, USA), followed by purification using DyeEx 2.0 spin purification kit (Qiagen, Hilden, Germany), and loaded for reading in the 3500xl Genetic Analyzer (Applied Biosystems Waltham, MA, USA).

The nucleotide sequences were then edited and aligned using Geneious Prime 2021.1.1 (https://www.geneious.com) based on the multiple sequence alignment tool, MAFFT (Multiple Alignment using Fast Fourier Transform). Sequences generated in this study were submitted to the Global Initiative on Sharing All Influenza Data (GISAID) database under accession numbers EPI1941118-25 and EPI1941139-63.

All available Egyptian H9N2 sequences (until October 2021), in addition to other representative viruses required, were retrieved from the public database (NCBI). Phylogenetic analysis was conducted by employing a maximum likelihood methodology based on the Akaike criterion after selecting the best fit modes using IQ-tree software version 1.1.3 [21]. Trees were finally viewed and edited using FigTree v1.4.4 software (http://tree.bio.ed.ac.uk/software/figtree/) (accessed on 5 October 2021) and Inkscape 1.1.

### 2.3. Ethical Approval

Three weeks old white Leghorn chickens were purchased from Nile SPF Farm, Kom Oshiem, Fayom, Egypt, and raised at RLQP-AHRI. Birds were kept in isolation units with daily feed and water. Bird experiments were legally approved by the Committee of Ethics of Animal Experiments at the AHRI, Egypt, under the protocol number AHRI-0249 and performed in strict accordance with the AHRI welfare and ethics guidelines. Infection experiments were performed in isolators at biosafety level 3 (BSL-3) animal facility. The study was performed in compliance with the ARRIVE guidelines https://arriveguidelines.org/arrive-guidelines (accessed on 2 March 2021).

### 2.4. Pathogenicity and Transmission

**Figure 1:**
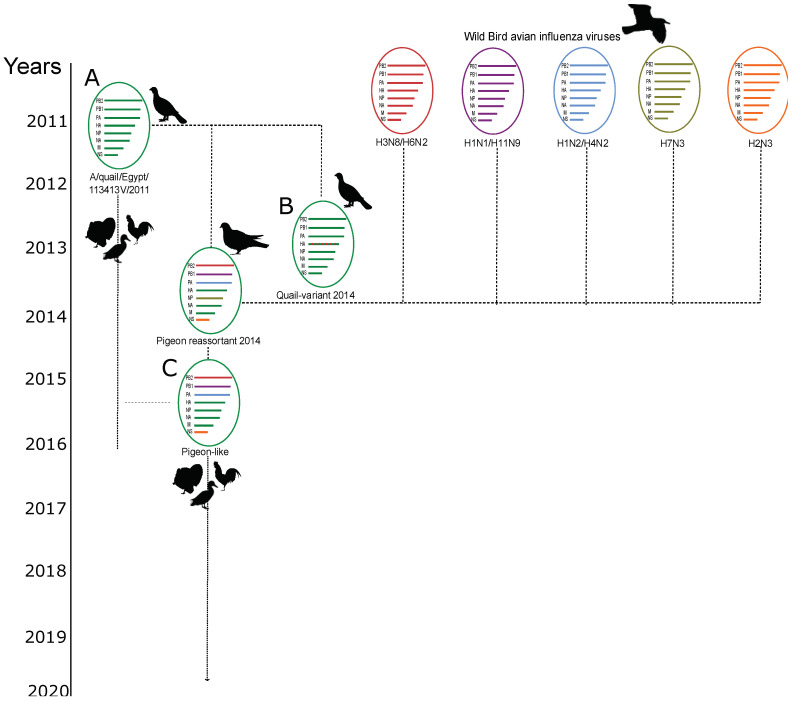
Different LPAI H9N2 viruses reported in Egypt (2011–2021). (**A**) The first reported LPAI H9N2 virus variant (A/quail/113413V/2011) in Egypt in 2011. (**B**) The quail/14864V/2014 variant detected in Egypt in 2014. (**C**) The recently circulating LPAI H9N2 variant in Egypt that harbors the *PB2, PB1, PA*, and NS genes from the pigeon variant and the rest of the genes from the Egyptian A/quail/113413V/2011-like variants. The three virus strains (**A**–**C**) were used for the chicken experiment. Silhouettes of duck (by Maija Karala) and Pigeon (by Dori (dori@merr.info) and Nevit Dilmento) were retrieved from PhyloPic (http://phylopic.org, accessed on 11 January 2022) under a CC BY-NC-SA-3.0 license (https://creativecommons.org/licenses/by-nc-sa/3.0/). Other silhouettes were downloaded from the same cite under a CC0 1.0 Universal (CC0 1.0) license (https://creativecommons.org/publicdomain/zero/1.0/).

Three Egyptian LPAI H9N2 virus variants with different genetic constitutions (Figure 1) were selected and compared for their pathogenicity: the ch/V1172/2018 variant represents the most recently circulating strain in Egypt (pigeon-like); the quail/14864V/2014 variant strain was isolated only from quails in 2014 and quail/113413V/2011 was the first detected LPAI H9N2 virus in Egypt.

Briefly, three groups of 3 weeks old chickens (10 in each group) were individually inoculated via the ocular-nasal route with 200 µL of EID50 = 10^7^/mL of the selected virus variants. In addition, a negative control group of 10 birds was inoculated with sterile phosphate-buffered saline (PBS). At 1 day post-infection (dpi), two sentinel chickens were introduced to each infected group to assess virus transmission. Chickens were monitored daily for clinical signs and mortality until 11 dpi. Cloacal (CL) and oropharyngeal (OP) swabs were collected at 1, 2, 4, 7, and 11 dpi. In addition, two chickens from each group were euthanized at 2, 4, and 7 dpi, and trachea, lung, spleen, pancreas, intestine (Ileocecal junction), kidney, and bursa of Fabricius were obtained for virus detection. The tissue samples were homogenized and centrifuged, and the supernatants were collected. Collected swabs and tissues were tested against influenza A virus *M* gene by RT-qPCR. Standard curves were created against a quantified reference virus to measure the virus concentration in the collected samples. Obtained data was analyzed and visualized using the ggplot2 package in R Version 4.0.2 [22,23]. Furthermore, the *p*-value for comparison between different groups was calculated by two-way ANOVA in RStudio version 1.4.1106, followed by a Tukey multiple pairwise comparison.

For histopathological examination, half of each trachea, lung, intestine, pancreas, spleen, bursa, and kidney was fixed in 10% neutral buffered formalin and processed by paraffin embedding techniques [24]. The scoring of histopathology was accomplished according to Landmann et al., 2021 [25]. Briefly, each organ was collected from birds in each group at 2, 4, and 7 dpi. Organs were examined for the different parameters shown in Tables S2 and S3, at five randomly selected microscope fields in each slide. Each field was evaluated according to the severity of histopathological lesion and scored from 0 to 3 using the following definition: 0 = no changes, 1 = mild, 2 = moderate, and 3 = severe. All scores were compared between groups, and statistically significant differences in severity between groups in each organ at each day of organ collection were assessed by one-way ANOVA.

### 2.5. Antigenic Characterization

Three viral antigens were generated and prepared from ch/V1172/2018, ch/V1410/2018, and ch/CAD74/2017 through inactivation with beta-propiolactone (BPL) at a concentration of 1:1000. The inactivated antigens were loaded on Montanide ISA 70 VG oil adjuvant (Seppic, France). A total volume of 500 µL of each antigen, containing 7 log2 (2560 HAU/dose), was inoculated subcutaneously into three 4-weeks old SPF chickens. A booster dose was administered after 2 weeks, blood samples were collected, and polyclonal antisera were retrieved 3 weeks later.

The prepared antigens and antisera of the viruses in our study were tested for antigenicity against homologous viruses and other LPAI H9N2 virus variants representing different sub-lineages of G1. The panel of cross antigenicity test included three recent Egyptian H9N2 virus variants and three earlier circulating Egyptian strains (quail/113413V/2011, quail/V864/2014, and ch/FAO-SL5/2015), which were prepared previously [10], as well as three antigens and antisera of various H9N2 genetic sub-lineages that Istituto Zooprofilattico Sperimentale delle Venezie kindly supplied from Italy (ch/Iran/10VIR/2008-G1/B, ch/KSA/VIR/2008-G1/A and mallard/Italy/2005: wild bird lineage) (Table S4). The antisera were heat-inactivated at 56 °C for 30 min, then a cross hemagglutination inhibition (HI) assay was applied on the different sera and antigens, according to the OIE manual for three successive repeats. Antigenic cartography analysis was performed using AntigenMap 3D server (https://sysbio.missouri.edu/AntigenMap, accessed on 15 May 2021) [26].

## 3. Results

### 3.1. Genetic and Antigenic Characterization

The whole-genome sequences of four Egyptian LPAI H9N2 virus variants were obtained for molecular characterization. The sequences were compared to other representative Egyptian LPAI H9N2 virus variants retrieved from the NCBI GenBank (2010–2021). Genetic similarities indicated that the sequenced strains harbored four genes (*HA*, *NP*, *NA*, *M*) from the ancestral Egyptian LPAI H9N2 virus with nucleotide similarities of 99.4–100. The remaining genes (*PB2*, *PB1*, *PA*, and *NS*) from all four sequenced virus strains were 95–99% identical to the nucleotide sequence of A/pigeon/Egypt/S10408B/2014 (accession No. KX000876). Sequence analyses of the HA revealed that all sequenced virus variants harbor basic amino acids cleavage sites (HACS) motif (-PARSSR/GLF-), indicating low pathogenicity. The amino acids at positions 226 and 228 of the HA of the sequenced virus strains were found to have leucine in position 226 (226L) and glycine in position 228 (228G). A switch from glutamine to leucine in position 226 (Q226L) is associated with increased mammalian-like terminal sialic acid affinity [27]. The NA in all sequenced isolates revealed no markers of oseltamivir antiviral resistance and harbor 119E, 198D, 222I, 274H, and 292R, as reported in previously described Egyptian LPAI H9N2 viruses [28]. Molecular screenings of *PB2*, *PB1*, *PA*, *M*, and *NS* discloses the avian-like profile. The PB2 of the recent Egyptian H9N2 virus variant (from 2015) carries 627E (associated with lower virulence in mammalian cells [29]), as compared to 627V in previous variants. Analysis of the M2 protein showed 27I, associated with amantadine resistance [30], in all sequenced isolates in this study, compared to 27V present in previously described Egyptian LPAI H9N2 viruses.

### 3.2. Phylogenetic Characterization

**Figure 2:**
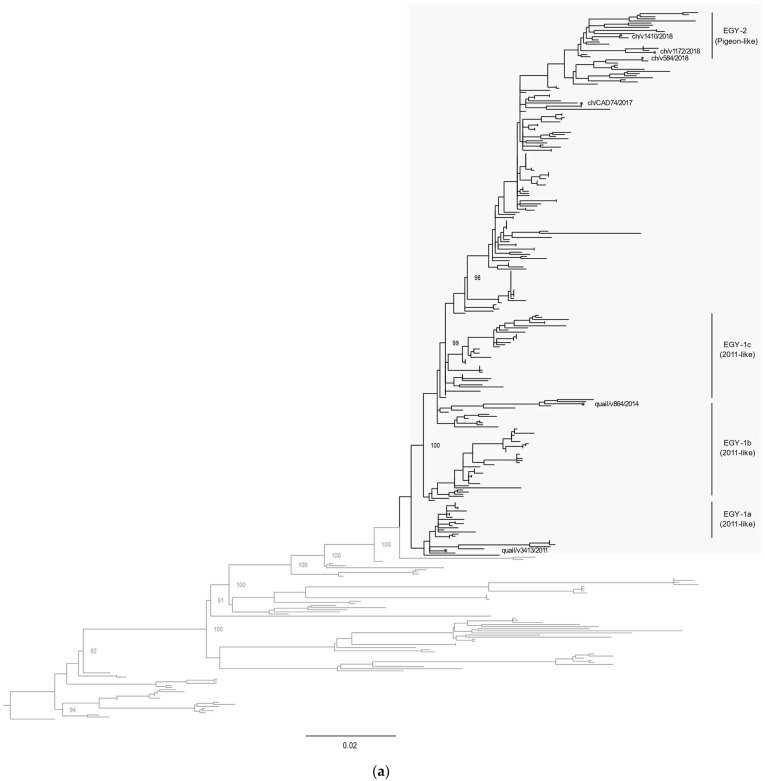

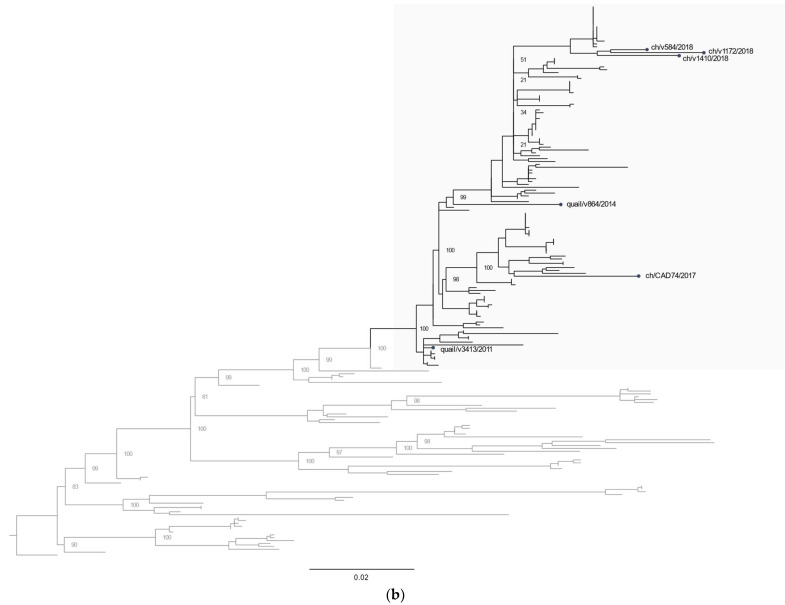
Phylogenetic trees of the nucleotide sequences of the *HA* (**a**) and *NA* (**b**) gene segments. The whole *HA* (**a**) and *NA* (**b**) gene segment were used from viruses reported till October 2021 and available at NCBI. Maximum likelihood calculations were done using the IQTree software under the best fit model, according to the Bayesian criterion. Egyptian LPAIV H9N2 virus variants are grey-shaded. Virus strains sequenced in this study are indicated by a black dot.

All eight gene segments of the sequenced virus strains were analyzed for phylogenetic relatedness. Phylogenetic analysis of the HA gene segments indicated that the Egyptian LPAI H9N2 virus variants clustered into several groups within the G1-B sub-lineage: EGY-1a–c (2011-like) and EGY-2 (pigeon-like). Virus strains of the EGY-2 group have been the most predominantly circulating since 2015, while there were no more reports of EGY-1 variants after 2016. The *HA* gene of ch/CAD74/2017, ch/v1172/2018, ch/v1410/2018, and ch/v584/2018 clustered with the EGY-2 (pigeon-like) (Figure 2a). Similar phylogenetic relatedness was found for the *NA* gene segment (Figure 2b). The *NP* and *M* gene segments were phylogenetically distinct from the same genes in viruses isolated from pigeon in 2014 (Supplementary Figure S1d,e). Phylogenetic analysis of the *PB2*, *PB1*, *PA*, and *NS* revealed clustering of ch/CAD74/2017, ch/v1172/2018, ch/v1410/2018, and ch/v584/2018 with the pigeon-like viruses (Figure S1a–c,f). These results suggest that the EGY-2 (pigeon-like) group has been the most commonly circulating LPAI H9N2 genotype in Egypt since 2016 (Figure 1).

### 3.3. Pathogenicity and Transmission in Chickens

**Figure 3:**
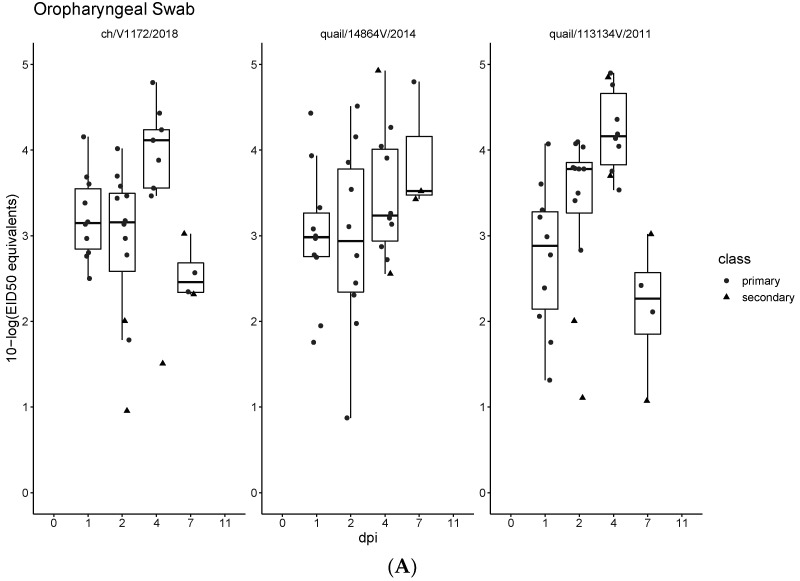

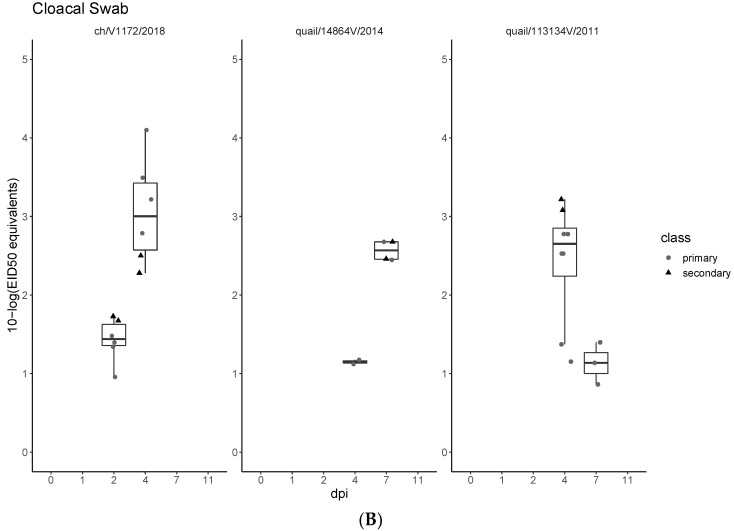
Virus shedding of LPAIV H9N2 infected chickens. Oropharyngeal (**A**) and cloacal (**B**) swabs were sampled at indicated days post-infection (dpi) and tested by RT-qPCR. Individual results of detected RNA copy numbers are given as EID_50_ equivalents. Circle indicates primary chicken and triangle indicates secondary/contact chickens.

Chickens were challenged with three of the four Egyptian LPAI H9N2 virus variants (ch/V1172/2018, quail/14864V/2014, and quail/113413V/2011) by oculonasal inoculation. Virus was detected in the oropharyngeal swab samples, starting from 1 dpi until 7 dpi in all groups. Viral shedding in cloacal swabs was detected at 2 dpi in the ch/V1172/2018 infected group (positive samples, *n* = 4) and starting from 4 dpi in quail/14864V/2014 (*n* = 2) and quail/113413V/2011 (*n* = 6) (Figure 3A,B), where the latter two continued till 7 dpi. In the second group of sentinel chickens, the virus was detected in both oropharyngeal and cloacal swab samples, starting from 1 dpi to 7 dpi. No virus was detected in the oropharyngeal or cloacal swab samples collected at 11 dpi in any of the groups. None of the infected birds showed any clinical signs of respiratory disease. All swabs collected from the negative control group were PCR negative. Virus RNA was detected in all collected organs in all infected groups (trachea, lung, spleen, pancreas, intestine (Ileocecal junction), kidney, and bursa of Fabricius), as shown in Figure S2.

Histologically, a normal architecture was observed in all negative control birds. However, in the infected birds, variable microscopic lesions were observed in examined organs at 2 dpi, and the severity of the lesions increased with time progression (Figures S3–S7 and Tables S3 and S4). All inoculated groups showed tracheal lesions that varied from mild tracheitis to severe necrotic lesions, which were observed early in the quail/14864V/2014 and quail/V3413/2011 infected groups (Figure S3). A similar pattern was seen in the intestines that displayed mild to moderate inflammation in early infection, then the severity progressed to severe enteritis and lymphocytic infiltration with vacuolation of the epithelial lining, ending with degeneration (Figure S4). Moreover, marked pathological alterations were observed in lungs (Figure S5) and spleen (Figure S6) in all three groups. Early prominent lesions of the bursa of Fabricius (Figure S7) were observed in the quail/113413V/2011 infected group, and this was characterized by marked lymphocytic depletion, while the two other groups showed milder alterations with time progression in the bursa. Statistically significant differences in severity scores (*p*-value ≤ 0.05) at different time points between groups infected with the different viruses were found mainly in bursa, trachea, and spleen (Table S3). However, none of the organs had consistent differences in severity scores between groups throughout the experiment period.

### 3.4. Cross Antigenicity

**Figure 4:**
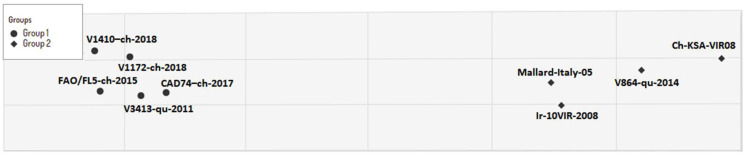
Antigenic cartography based on the hemagglutination inhibition (HI) titers of the sera of the Egyptian H9N2 virus strains used in this study and representative virus isolates of different antigenic clusters. Each point indicates a virus and both axes represent antigenic distance. The spacing between grid lines represents 1 antigenic unit distance, corresponding to a 2-fold dilution in the HI assay.

According to the antigenic (antibody) reactivities, the virus variants could be divided into two groups (Figure 4): group-1 included five Egyptian virus variants that circulated from 2011 to 2018 (V3413-qu-2011, FAO/FL5-ch-2015, CAD74-ch-2017, v1172-ch-2018, and v1410-ch-2018), while group-2 contained the Egyptian quail variant quail/14864V/2014 and three heterogeneous virus variants from different H9N2 sub-lineages of G1 (Ir-10VIR-2008-G1/B, ch-KSA-VIR08-G1/A, and mallard-Italy-05).

## 4. Discussion

Avian influenza viruses of the subtype H9N2 continue to cause economic losses to poultry production worldwide and have become the dominant enzootic subtype in chicken farms in several countries [1]. In mid-2011, LPAI H9N2 virus was detected in a quail farm for the first time in Egypt and, in a short time, affected several poultry farms all over Egypt [5]. Several reports have demonstrated increased pathogenicity of LPAI H9N2 in chickens, mainly accompanied by co-infection with other viruses or bacterial pathogens [31]. Furthermore, LPAI H9N2 virus variants have been involved in reassortment events with both wild bird virus strains and strains from domestic poultry [11,32]. In the last 10 years, the phylogenetic analyses of Egyptian LPAI H9N2 virus strains have revealed clustering into three groups (a–c) within the G1-B sub-lineage [33]. Intra- and inter-subtypic reassortment have been documented within the Egyptian H9N2 virus variants [11,14,32]. The variation in antigenicity and pathogenicity among those different genotypes of Egyptian H9N2 has been unclear. Therefore, we aimed to genetically and antigenically characterize candidate strains of different genotypes detected in Egypt and determine their pathogenicity.

Our results revealed that the LPAI H9N2 virus variants that harbor the *PB2*, *PB1*, *PA*, and *NS* gene segments from the pigeon/2014 H9N2 strain have been circulating since 2014 and have become the dominant H9N2 genotype circulating in Egypt from 2016 to 2021 (Figure 1 and Figure 2). None of the infected chickens showed any symptoms of respiratory disease, but virus shedding was detected up to 7 dpi in all infected groups (although no virus was detected at 11 dpi). No statistically significant difference in virus shedding between the infected groups was found. However, a considerable variation in virus shedding at day 7 dpi was detected, where the quail/14864V/2014 showed an increased virus shedding in both tracheal and cloacal swabs at 7 dpi. The LPAI viruses are described as mainly targeting the respiratory tract, limitedly targeting the gastrointestinal tract, and usually associated with poor cloacal virus shedding in infected chickens [34,35]. It was noticed that there are different patterns in cloacal shedding among the infected groups, where the ch/V1172/2018 virus produced earlier cloacal virus shedding at 2 dpi to 4 dpi compared to the other two viruses, which started CL virus shedding on 4 dpi till 7 dpi. Variation in the virus shedding onset and the duration of virus shedding was described in previous studies with LPAI viruses [35,36].

This indicated the ability of the Egyptian LPAI H9N2 variants to infect both the respiratory and the digestive tract. A previous study revealed that other H9N2 virus variants of the Y439-like lineage could be detected until 7 dpi intranasally in infected chickens with no clinical signs [37]. Normal histological architecture was observed in all birds in the negative control group. Variable microscopic lesions were observed in infected organs at 2 dpi, and the severity increased with time progression. All infected groups showed variable tracheal lesions that varied from mild tracheitis to severe necrotic lesions, which was observed early in the groups infected with quail/14864V/2014 and quail/113413V/2011. Moreover, marked pathological alterations were observed in lungs of all groups, but no difference in intensity between groups was noticed. In addition to the ability of H9N2 virus variants to induce infection in the respiratory and digestive tracts, the lymphoid organs were also affected. Bursa and spleen suffered from lymphocytic depletion and degenerative reactions with different degrees of severity among the infected groups. Additionally, the inoculated virus was detected in both organs until 7 dpi. The impact of H9N2 on the lymphoid tissues has been reported previously both in the Egyptian strains and various strains related to other lineages [38,39]. Our antigenic characterization of both the current and the 2011-like variants revealed that they are still antigenically homologous. On the contrary, the quail/14864V/2014 is still antigenic heterogeneous compared to the other Egyptian variants, as shown in previous studies [10,11].

Our study extends the current understanding of the genetic and pathogenic characteristics of LPAI H9N2 virus variants in Egypt. Thus, it is essential to strengthen the surveillance of AIV in both wild and domestic poultry coupled with whole-genome sequencing to continue detailed monitoring of the virus evolution and to detect novel reassortant influenza viruses.

## Data Availability

The nucleotide sequences generated in this paper have been deposited into a public database: Global Initiative on Sharing All Influenza Data (GISAID) database under accession numbers shown in Table S1.

## Supplementary Materials

The following are available online at https://www.mdpi.com/article/10.3390/v14051030/s1, Figure S1: Phylogenetic tree of the internal gene segments of Egyptian H9N. Figure S1a: Phylogenetic tree of the *PB2* gene segment; Figure S1b: Phylogenetic tree of *PB1* gene segment; Figure S1c: Phylogenetic tree of *PA* gene segment; Figure S1d: Phylogenetic tree of *NP* gene segment; Figure S1e: Phylogenetic tree of *M* gene segment; Figure S1f: Phylogenetic tree of *NS* gene segment; Figure S2: Virus detection in collected tissues samples 2, 4, and 7 days post-infection; Figure S3: Pathological changes in trachea; Figure S4: Pathological changes in intestine; Figure S5: Pathological changes in the lung; Figure S6: Pathological changes of the spleen; Figure S7: Pathological changes of bursa; Table S1: H9N2 viruses isolated and sequenced in this study; Table S2: Histopathological findings for three groups of chickens infected: Ch/V1171/2018 (Group 1), Quail/14864V/2014 (Group 2), Quail/113413V/1 2011 (Group 3); Table S3: Quantitative Scoring of histopathological changes in examined organs (data are shown as mean ± SD); Table S4: Antigenic characterization of G1 lineage H9N2 viruses.

## References

[1] Peacock T.H.P., James J., Sealy J.E., Iqbal M. (2019). A Global Perspective on H9N2 Avian Influenza Virus. Viruses.

[2] Sun Y., Liu J. (2015). H9N2 influenza virus in China: A cause of concern. Protein Cell.

[3] Kalonda A., Saasa N., Nkhoma P., Kajihara M., Sawa H., Takada A., Simulundu E. (2020). Avian Influenza Viruses Detected in Birds in Sub-Saharan Africa: A Systematic Review. Viruses.

[4] Song W., Qin K. (2020). Human-infecting influenza A (H9N2) virus: A forgotten potential pandemic strain?. Zoonoses Public Health.

[5] Li R., Adel A., Bohlin J., Lundkvist Å., Olsen B., Pettersson J.H., Naguib M.M. (2020). Phylogeographic Dynamics of Influenza A(H9N2) Virus Crossing Egypt. Front. Microbiol..

[6] Steel J., Lowen A.C. (2014). Influenza A virus reassortment. Curr. Top. Microbiol. Immunol..

[7] Guan Y., Shortridge K.F., Krauss S., Webster R.G. (1999). Molecular characterization of H9N2 influenza viruses: Were they the donors of the “internal” genes of H5N1 viruses in Hong Kong?. Proc. Natl. Acad. Sci. USA.

[8] Chang H.P., Peng L., Chen L., Jiang L.F., Zhang Z.J., Xiong C.L., Zhao G.M., Chen Y., Jiang Q.W. (2018). Avian influenza viruses (AIVs) H9N2 are in the course of reassorting into novel AIVs. J. Zhejiang Univ. Sci. B.

[9] El-Zoghby E.F., Arafa A.S., Hassan M.K., Aly M.M., Selim A., Kilany W.H., Selim U., Nasef S., Aggor M.G., Abdelwhab E.M. (2012). Isolation of H9N2 avian influenza virus from bobwhite quail (Colinus virginianus) in Egypt. Arch. Virol..

[10] Adel A., Arafa A., Hussein H.A., El-Sanousi A.A. (2017). Molecular and antigenic traits on hemagglutinin gene of avian influenza H9N2 viruses: Evidence of a new escape mutant in Egypt adapted in quails. Res. Vet. Sci..

[11] Kandeil A., El-Shesheny R., Maatouq A., Moatasim Y., Cai Z., McKenzie P., Webby R., Kayali G., Ali M.A. (2017). Novel reassortant H9N2 viruses in pigeons and evidence for antigenic diversity of H9N2 viruses isolated from quails in Egypt. J. Gen. Virol..

[12] Hassan K.E., Saad N., Abozeid H.H., Shany S., El-Kady M.F., Arafa A., El-Sawah A.A.A., Pfaff F., Hafez H.M., Beer M. (2020). Genotyping and reassortment analysis of highly pathogenic avian influenza viruses H5N8 and H5N2 from Egypt reveals successive annual replacement of genotypes. Infect. Genet. Evol..

[13] Naguib M.M., El-Kady M.F., Lüschow D., Hassan K.E., Arafa A.S., El-Zanaty A., Hassan M.K., Hafez H.M., Grund C., Harder T.C. (2017). New real time and conventional RT-PCRs for updated molecular diagnosis of infectious bronchitis virus infection (IBV) in chickens in Egypt associated with frequent co-infections with avian influenza and Newcastle Disease viruses. J. Virol. Methods.

[14] Hagag N.M., Erfan A.M., El-Husseiny M., Shalaby A.G., Saif M.A., Tawakol M.M., Nour A.A., Selim A.A., Arafa A.S., Hassan M.K. (2019). Isolation of a Novel Reassortant Highly Pathogenic Avian Influenza (H5N2) Virus in Egypt. Viruses.

[15] Adel A., Mosaad Z., Shalaby A.G., Selim K., Samy M., Abdelmagid M.A., Hagag N.M., Arafa A.S., Hassan W.M., Shahien M.A. (2021). Molecular evolution of the hemagglutinin gene and epidemiological insight into low-pathogenic avian influenza H9N2 viruses in Egypt. Res. Vet. Sci..

[16] OIE (2021). Avian Influenza (Including Infection with Highly Pathogenic Avian Influenza Viruses).

[17] Meir R., Maharat O., Farnushi Y., Simanov L. (2010). Development of a real-time TaqMan RT-PCR assay for the detection of infectious bronchitis virus in chickens, and comparison of RT-PCR and virus isolation. J. Virol. Methods.

[18] Spackman E., Senne D.A., Myers T.J., Bulaga L.L., Garber L.P., Perdue M.L., Lohman K., Daum L.T., Suarez D.L. (2002). Development of a real-time reverse transcriptase PCR assay for type A influenza virus and the avian H5 and H7 hemagglutinin subtypes. J. Clin. Microbiol..

[19] Wise M.G., Suarez D.L., Seal B.S., Pedersen J.C., Senne D.A., King D.J., Kapczynski D.R., Spackman E. (2004). Development of a real-time reverse-transcription PCR for detection of newcastle disease virus RNA in clinical samples. J. Clin. Microbiol..

[20] Naguib M.M., Arafa A.S., El-Kady M.F., Selim A.A., Gunalan V., Maurer-Stroh S., Goller K.V., Hassan M.K., Beer M., Abdelwhab E.M. (2015). Evolutionary trajectories and diagnostic challenges of potentially zoonotic avian influenza viruses H5N1 and H9N2 co-circulating in Egypt. Infect. Genet. Evol..

[21] Minh B.Q., Schmidt H.A., Chernomor O., Schrempf D., Woodhams M.D., von Haeseler A., Lanfear R. (2020). IQ-TREE 2: New Models and Efficient Methods for Phylogenetic Inference in the Genomic Era. Mol. Biol. Evol..

[22] Wickham H. (2016). ggplot2: Elegant Graphics for Data Analysis.

[23] Therneau T.M., Grambsch P.M. (2000). Modeling Survival Data: Extending the Cox Model.

[24] Rhodes A., Suvarna S.K., Layton C., Bancroft J.D. (2013). Fixation of tissues. Bancroft’s Theory and Practice of Histological Techniques.

[25] Landmann M., Scheibner D., Graaf A., Gischke M., Koethe S., Fatola– O.I., Raddatz B., Mettenleiter T.C., Beer M., Grund C. (2021). A Semiquantitative Scoring System for Histopathological and Immunohistochemical Assessment of Lesions and Tissue Tropism in Avian Influenza. Viruses.

[26] Barnett J.L., Yang J., Cai Z., Zhang T., Wan X.-F. (2012). AntigenMap 3D: An online antigenic cartography resource. Bioinformatics.

[27] Sorrell E.M., Wan H., Araya Y., Song H., Perez D.R. (2009). Minimal molecular constraints for respiratory droplet transmission of an avian-human H9N2 influenza A virus. Proc. Natl. Acad. Sci. USA.

[28] Adel A., Mady W., Mosad Z., Amer F., Shaaban A., Said D., Maged M., Arafa A., Morsi M., Hassan M. (2019). Molecular and epidemiological overview on low pathogenic avian influenza H9N2 in Egypt between 2015 and 2016. Hosts Viruses.

[29] Paterson D., te Velthuis A.J., Vreede F.T., Fodor E. (2014). Host restriction of influenza virus polymerase activity by PB2 627E is diminished on short viral templates in a nucleoprotein-independent manner. J. Virol..

[30] Lee J., Song Y.J., Park J.H., Lee J.H., Baek Y.H., Song M.S., Oh T.K., Han H.S., Pascua P.N., Choi Y.K. (2008). Emergence of amantadine-resistant H3N2 avian influenza A virus in South Korea. J. Clin. Microbiol..

[31] Samy A., Naguib M.M. (2018). Avian Respiratory Coinfection and Impact on Avian Influenza Pathogenicity in Domestic Poultry: Field and Experimental Findings. Vet. Sci..

[32] Hassan K.E., King J., El-Kady M., Afifi M., Abozeid H.H., Pohlmann A., Beer M., Harder T. (2020). Novel Reassortant Highly Pathogenic Avian Influenza A(H5N2) Virus in Broiler Chickens, Egypt. Emerg. Infect. Dis..

[33] Naguib M.M., Arafa A.S., Parvin R., Beer M., Vahlenkamp T., Harder T.C. (2017). Insights into genetic diversity and biological propensities of potentially zoonotic avian influenza H9N2 viruses circulating in Egypt. Virology.

[34] Chrzastek K., Leng J., Zakaria M.K., Bialy D., La Ragione R., Shelton H. (2021). Low pathogenic avian influenza virus infection retards colon microbiota diversification in two different chicken lines. Anim. Microbiome.

[35] Spickler A.R., Trampel D.W., Roth J.A. (2008). The onset of virus shedding and clinical signs in chickens infected with high-pathogenicity and low-pathogenicity avian influenza viruses. Avian Pathol..

[36] van der Goot J.A., de Jong M.C., Koch G., Van Boven M. (2003). Comparison of the transmission characteristics of low and high pathogenicity avian influenza A virus (H5N2). Epidemiol. Infect..

[37] Kye S.J., Park M.J., Kim N.Y., Lee Y.N., Heo G.B., Baek Y.K., Shin J.I., Lee M.H., Lee Y.J. (2021). Pathogenicity of H9N2 low pathogenic avian influenza viruses of different lineages isolated from live bird markets tested in three animal models: SPF chickens, Korean native chickens, and ducks. Poult. Sci..

[38] Bano S., Naeem K., Malik S.A. (2003). Evaluation of pathogenic potential of avian influenza virus serotype H9N2 in chickens. Avian Dis..

[39] Song Y., Zhang Y., Chen L., Zhang B., Zhang M., Wang J., Jiang Y., Yang C., Jiang T. (2019). Genetic Characteristics and Pathogenicity Analysis in Chickens and Mice of Three H9N2 Avian Influenza Viruses. Viruses.

