# Supplementary material for: Genetic Variations among Different Variants of G1-like Avian Influenza H9N2 Viruses and Their Pathogenicity in Chickens
Source: Viruses. 2022 May 11;14(5):1030. doi: 10.3390/v14051030 (PMC9143995; doi:10.3390/v14051030)
Supplement: Supplementary file 1 [file viruses-14-01030-s001.zip › viruses-1701714-supplementary.pdf]

Supplementary Information for:

Genetic Variations among Different Variants of G1-like Avian Influenza H9N2 Viruses and Their Pathogenicity in Chickens

Amany Adel[1], Marwa A. Abdelmagid[1], Ahmed Abd-Elhalem Mohamed[1], Anishia Wasberg[2], Zienab Mosaad[1], Karim Selim[1], Asmaa Shaaban[1], Mohamed Tarek[1], Naglaa M. Hagag[1], Åke Lundkvist[2], Patrik Ellström[3], Mahmoud M. Naguib[1, 2, #]

[1]Reference Laboratory for Veterinary Quality Control on Poultry Production, Animal Health Research Institute, Agriculture Research Center, Giza 12618, Egypt

[2]Zoonosis Science Center, Department of Medical Biochemistry and Microbiology, Uppsala University, 751 21 Uppsala, Sweden

[3]Zoonosis Science Center, Department of Medical Sciences, Uppsala University, Uppsala SE-75185, Sweden

*Correspondence: Mahmoud M. Naguib Mahmoud.naguib@imbim.uu.se

Supplementary Figure S1. Phylogenetic tree of the internal gene segments of Egyptian H9N2. 1.a Phylogenetic tree of the PB2 gene segment. Virus strains sequenced in this study are colored in blue and reference strains are colored in red.

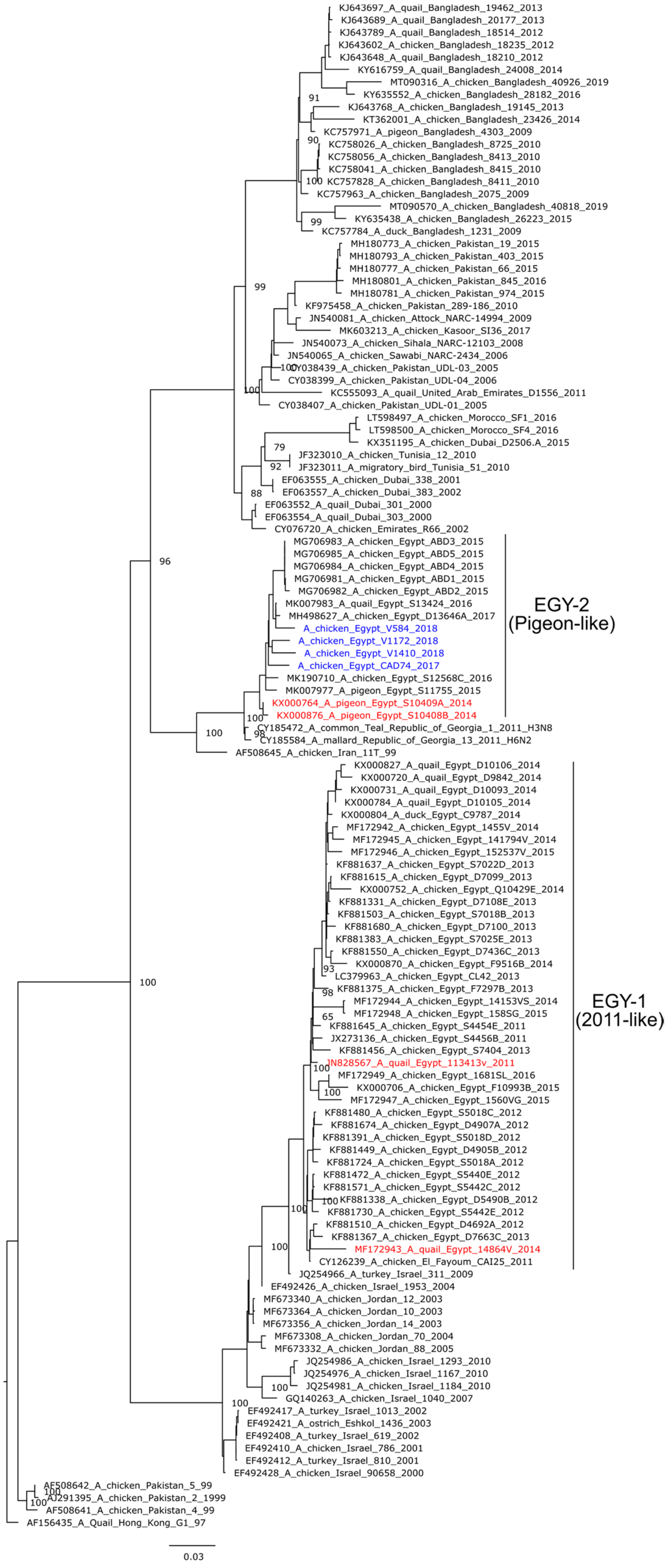

Supplementary Figure S1. b Phylogenetic tree of PB1 gene segment. Viruses sequenced in this study are colored in blue and reference strains are colored in red.

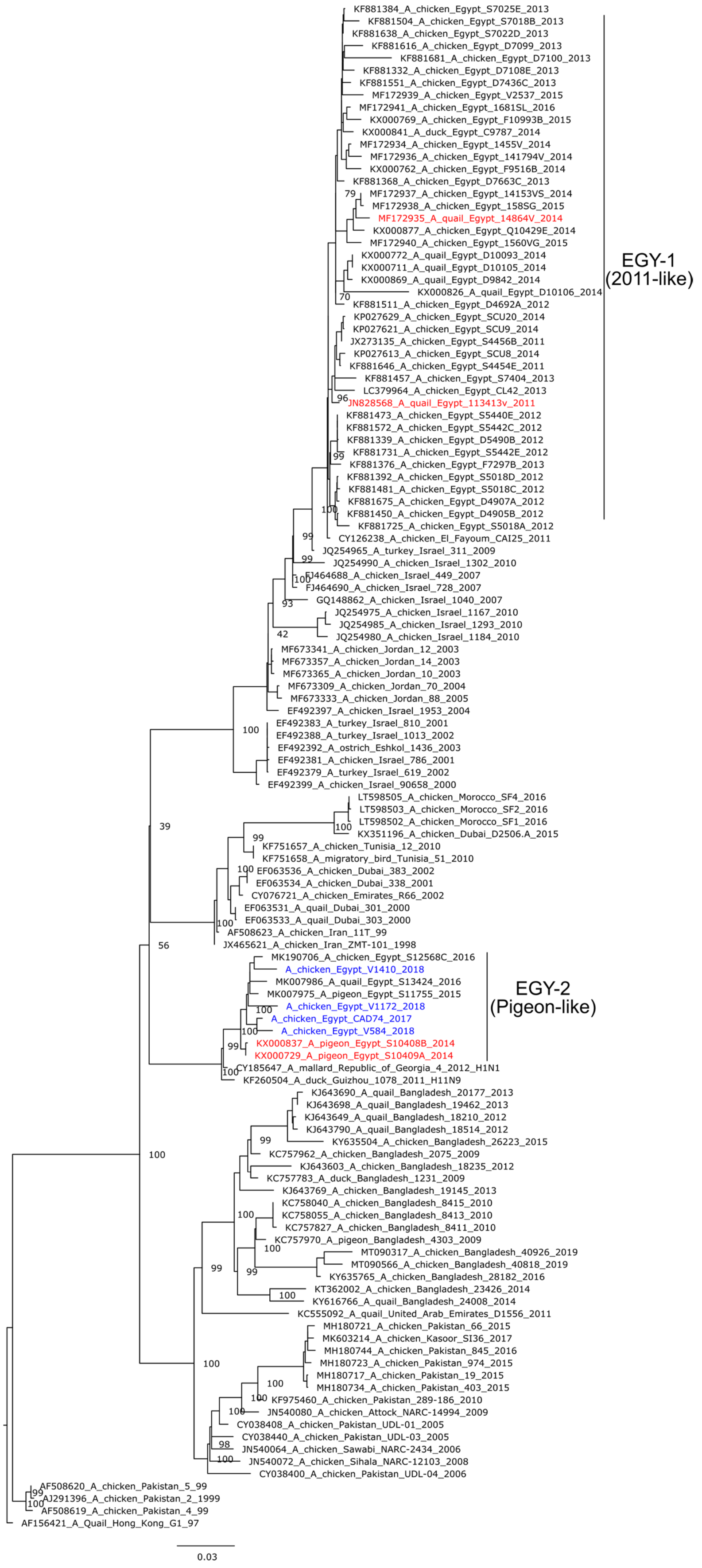

Supplementary Figure S1. c Phylogenetic tree of PA gene segment. Viruses sequenced in this study are colored in blue and reference strains are colored in red.

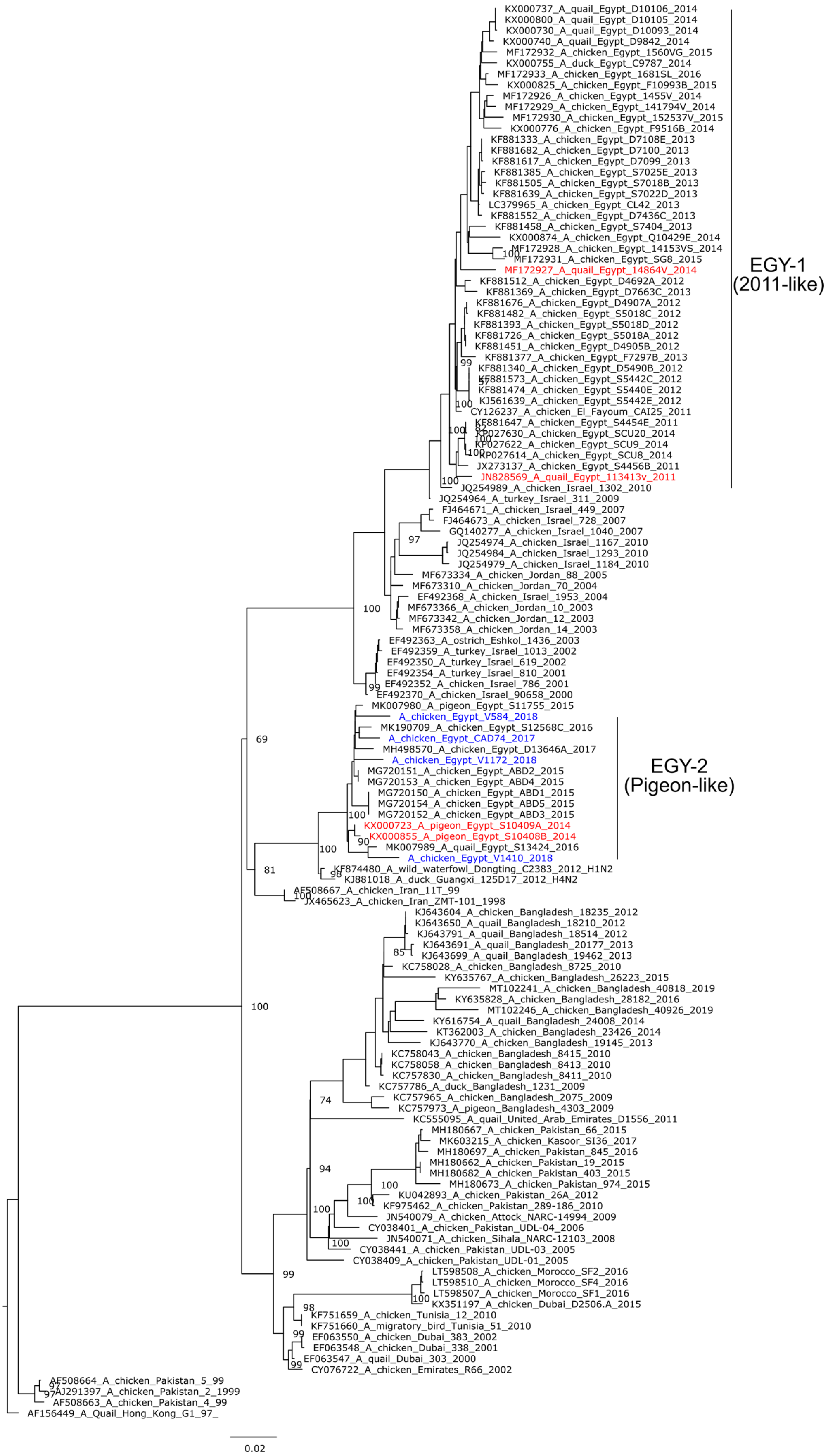

Supplementary Figure S1.d Phylogenetic tree of NP gene segment. Viruses sequenced in this study are colored in blue and reference strains are colored in red.

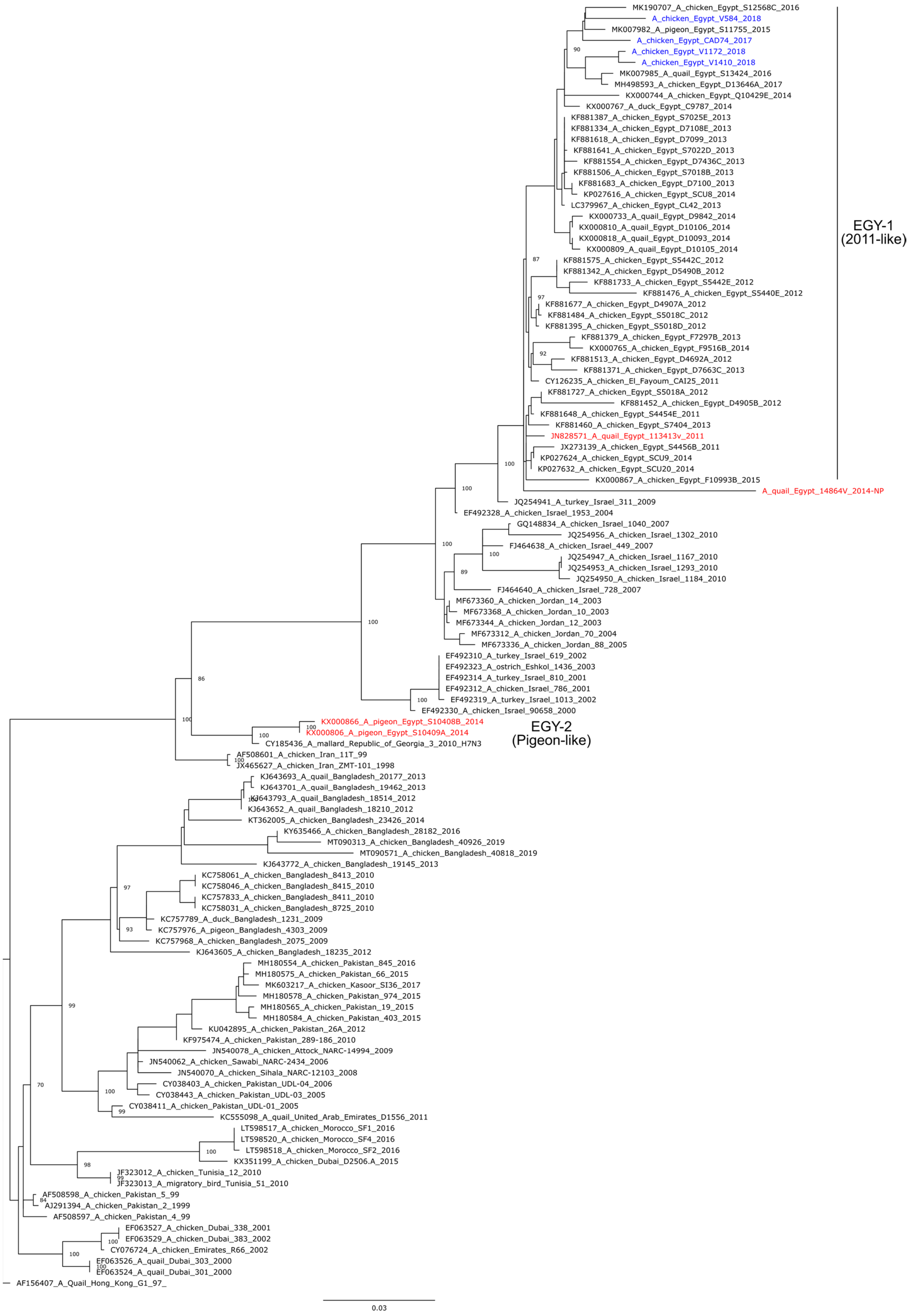

Supplementary Figure S1. e Phylogenetic tree of M gene segment. Viruses sequenced in this study are colored in blue and reference strains are colored in red.

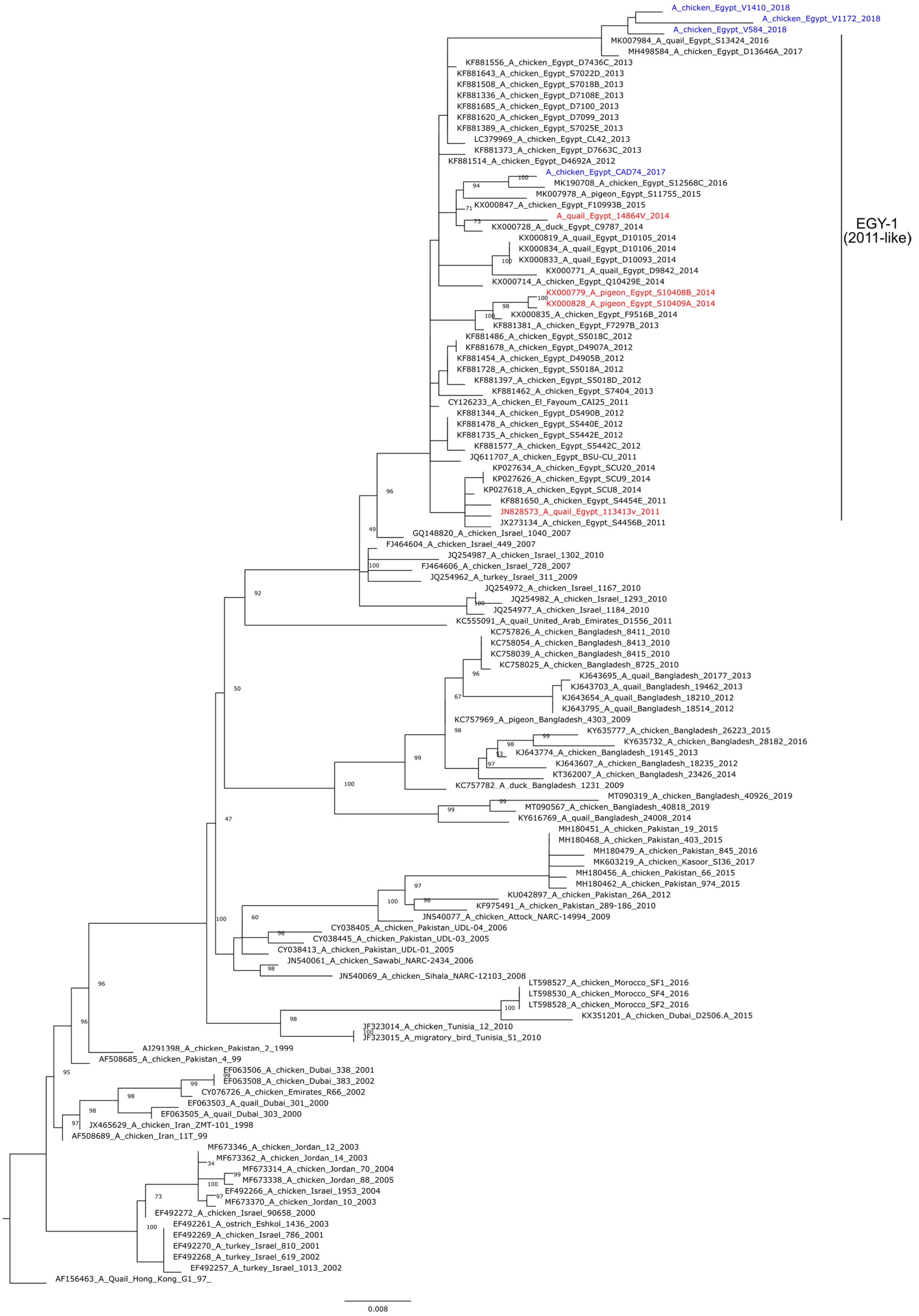

Supplementary Figure S1. f Phylogenetic tree of NS gene segment. Viruses sequenced in this study are colored in blue and reference strains are colored in red.

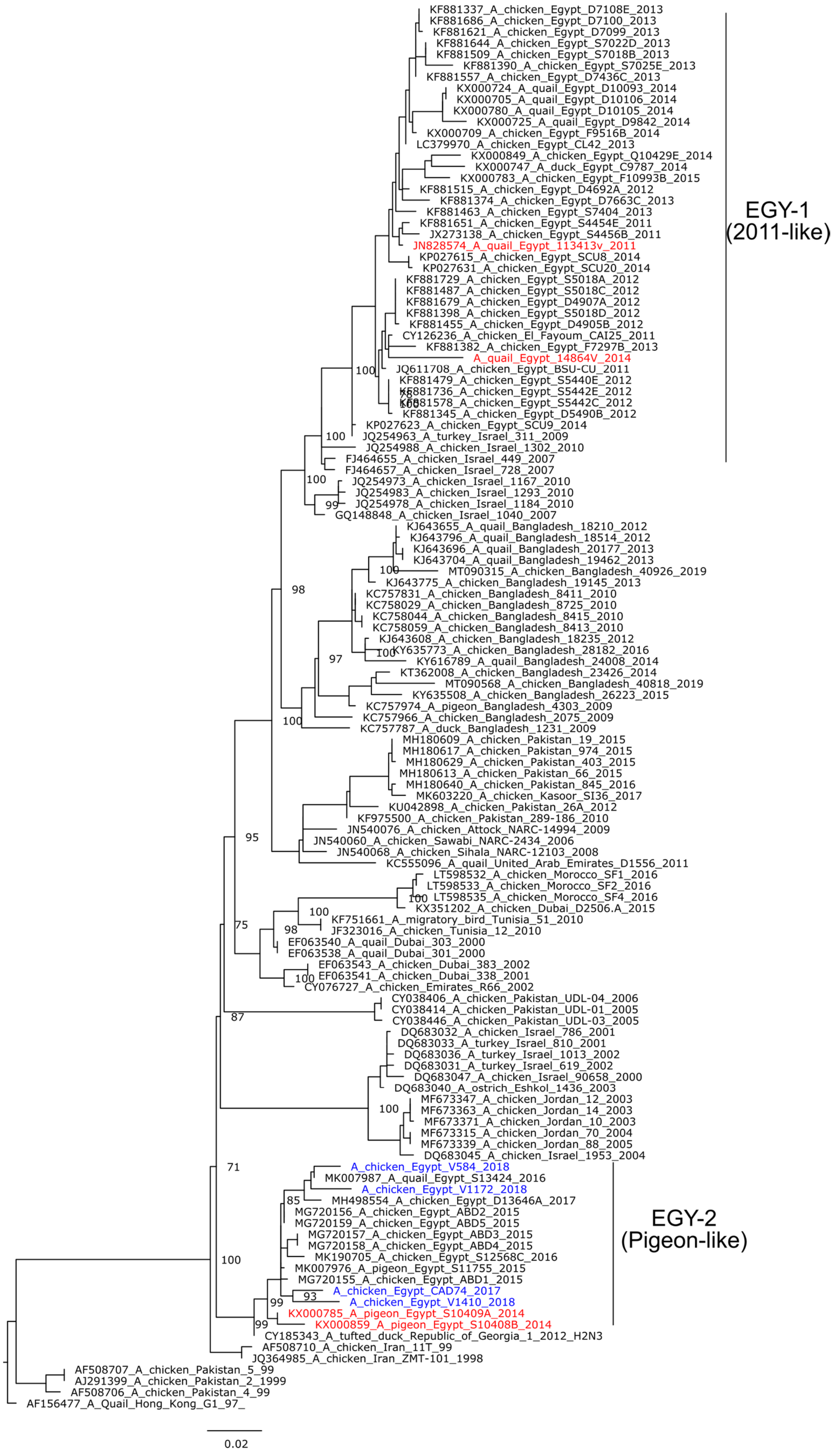

Supplementary Figure S2. Virus detection in collected tissues samples 2, 4 and 7 days post-infection.

Collected tissues were obtained at the indicated number of days post-infection (dpi) and tested by RT-qPCR. Individual results of detected RNA copy numbers are given as EID_{50} equivalents.

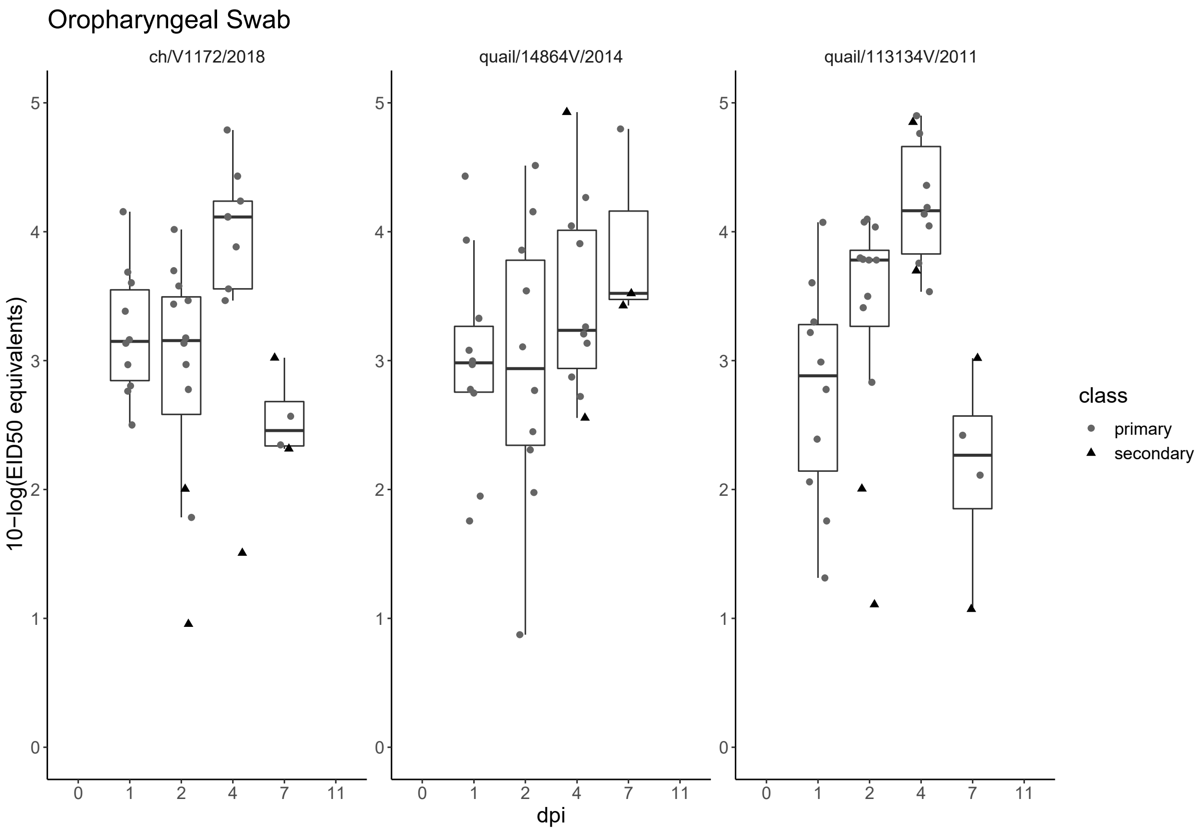

Supplementary Figure S3. Pathological changes in trachea

Ch/V1171/2018: normal architecture of trachea (1), mild tracheitis with marked submucosal lymphocytic cells infiltration (arrow) (2), severe tracheitis with complete necrosed lining epithelium (arrow) replaced by lymphocytic cells (3). **Quail/14864V/2014**: mild tracheitis with activation of the mucous gland (arrow) (1), necrosed lining epithelium replaced by lymphocytic cells (arrow) (2), marked submucosal lymphocytic cells infiltration and congested blood vessels (arrow) (3). **Quail/113134V/2011**: marked activation of mucous glands (arrow) and submucosal edema (red arrow) (1), submucosal hemorrhages and lymphocytic cells infiltration (arrow) (2), severe tracheitis with hyperplasia of epithelial surface and severe lymphocytic cells infiltration (arrow) (3). **H&E (X200)**

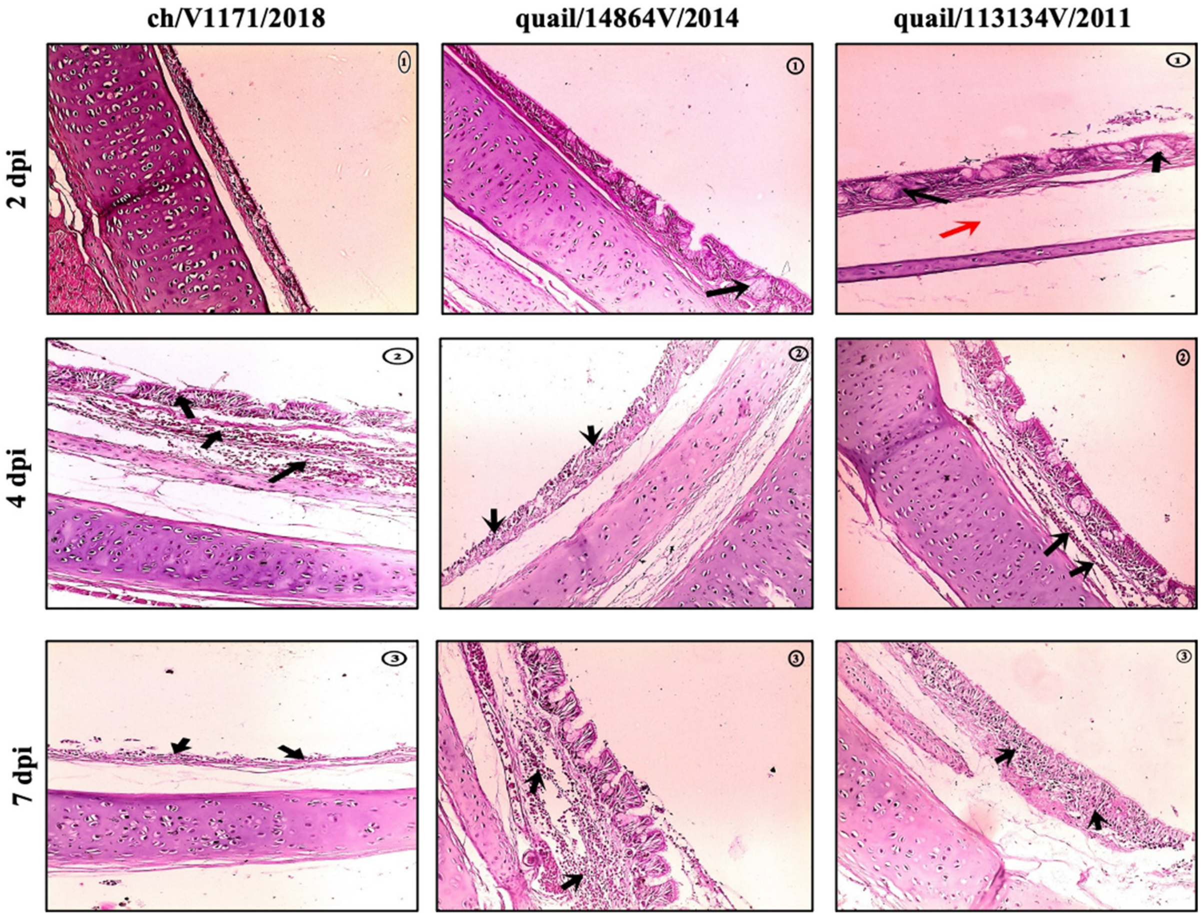

Supplementary Figure S4. Pathological changes in intestine.

Ch/V1171/2018: mild enteritis with mild lymphocytic cells infiltration (1), severe enteritis with mononuclear cells infiltration and vacuolation of epithelial lining and hyperplasia of intestinal glands (2), severe enteritis with degeneration of lining epithelial and severe lymphocytic cells infiltration (3). **Quail/14864V/2014**: moderate enteritis and vacuolation of epithelial lining and lymphocytic cells infiltration (red arrow) (1), severe enteritis with mononuclear cells infiltration (2), severe enteritis with degeneration of lining epithelial and severe lymphocytic cells infiltration (3). **Quail/113134V/2011**: moderate enteritis with vacuolation of epithelial lining and necrosis (1), severe enteritis with mononuclear cells infiltration and vacuolation of epithelial lining and hyperplasia of intestinal glands and thickening of muscular layer (2), severe enteritis with degeneration of lining epithelial and severe lymphocytic cells infiltration (3). **H&E (X200)**

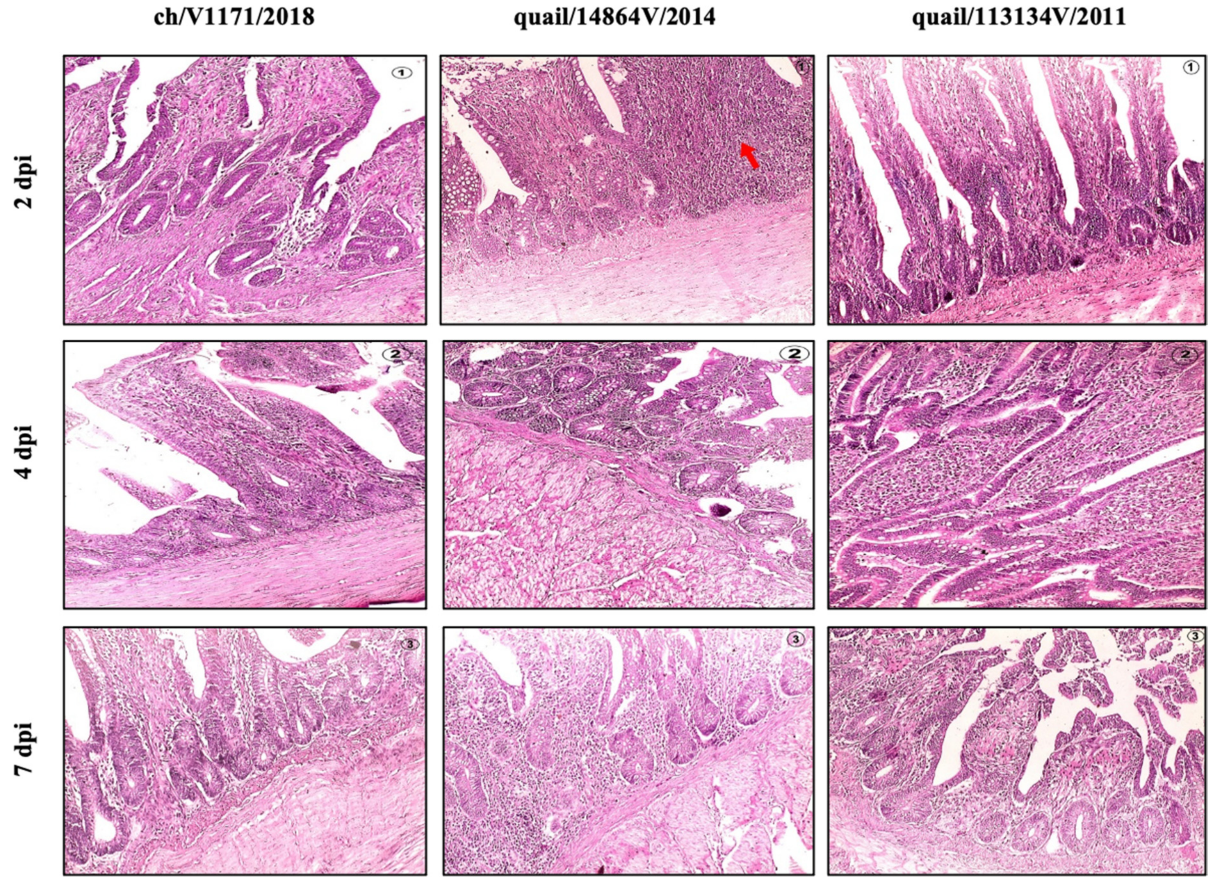

Supplementary Figure S5. Pathological changes in the lung.

Ch/V1171/2018: mild congestion of pulmonary blood vessels (1), mild pneumonia with lymphocytic cells infiltrations (arrow) and fibrous tissue proliferation (red arrow)(2), severe pneumonia with prominent thickening of the alveolar wall (red arrow) and lymphocytic cells infiltrations within the parenchyma (arrow) (3). **Quail/14864V/2014**: mild pneumonia with lymphocytic cells infiltration inside pulmonary blood vessels (arrow) (1), severe pneumonia with marked vasculitis and thrombus formation (red arrow) in addition to lymphocytic cells infiltration (arrow) (2), fibrinous pneumonia (arrow) with the formation of compensatory alveoli. **Quail/113134V/2011**: congestion of pulmonary blood vessels and lymphocytic cells infiltration (arrow) (1), marked hemorrhages within the parenchyma and 3ry bronchi mixed with mononuclear cells infiltration (arrow) (2), noticeable vasculitis and pneumonia (3). **H&E (X200)**

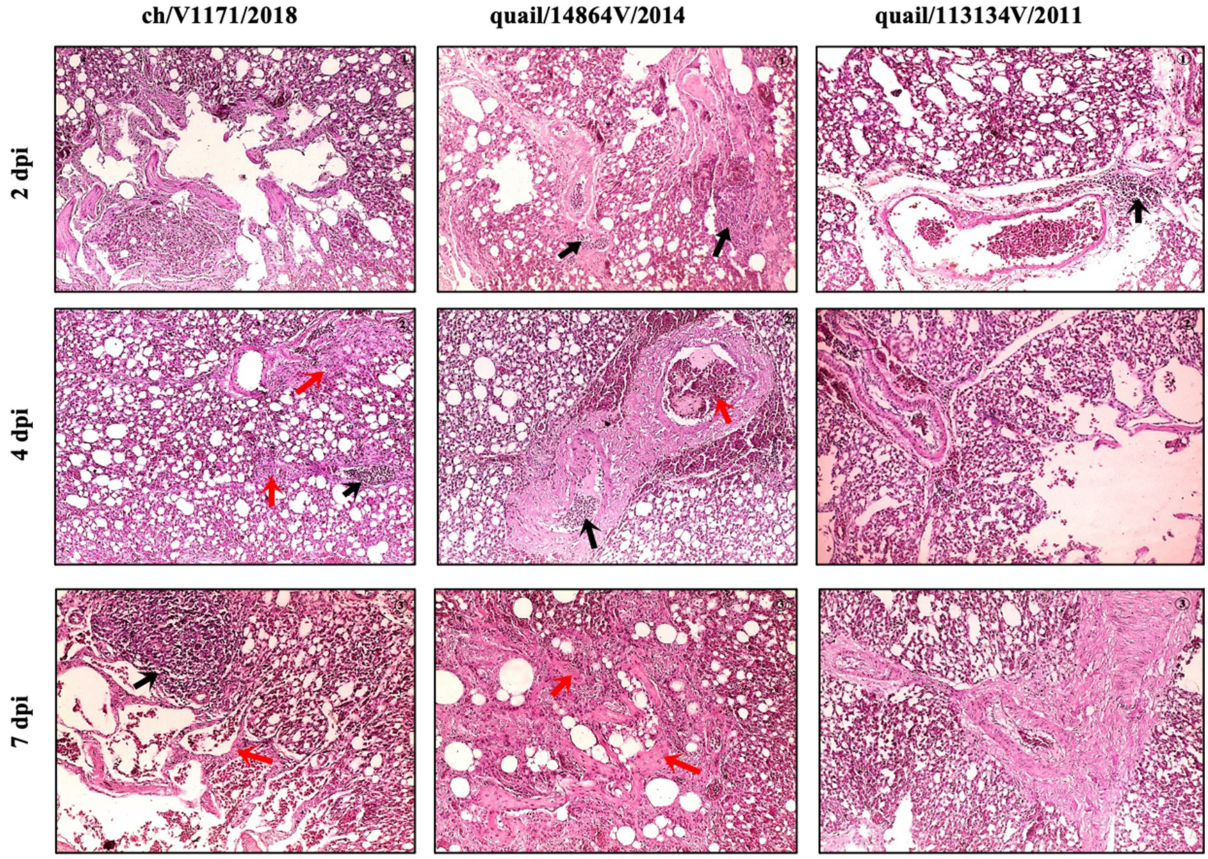

Supplementary Figure S6. Pathological changes of the spleen.

Ch/V1171/2018: normal architecture of spleen (1), mild lymphocytic depletion (arrow) (2), severe splenitis with multiple vasculitis (arrow) (3). **Quail/14864V/2014**: normal histological appearance (1), mild lymphocytic depletion (arrow) (2), moderate splenitis with lymphocytic depletion and heterophilic cells infiltration (arrow)(3). **Quail/113134V/2011**: mild lymphocytic depletion (1); splenitis with necrosed lymphocytic cells (arrow) (2), severe splenitis with noticeable vasculitis (arrow) and necrosed lymphocytic cells (3). **H&E (X200)**

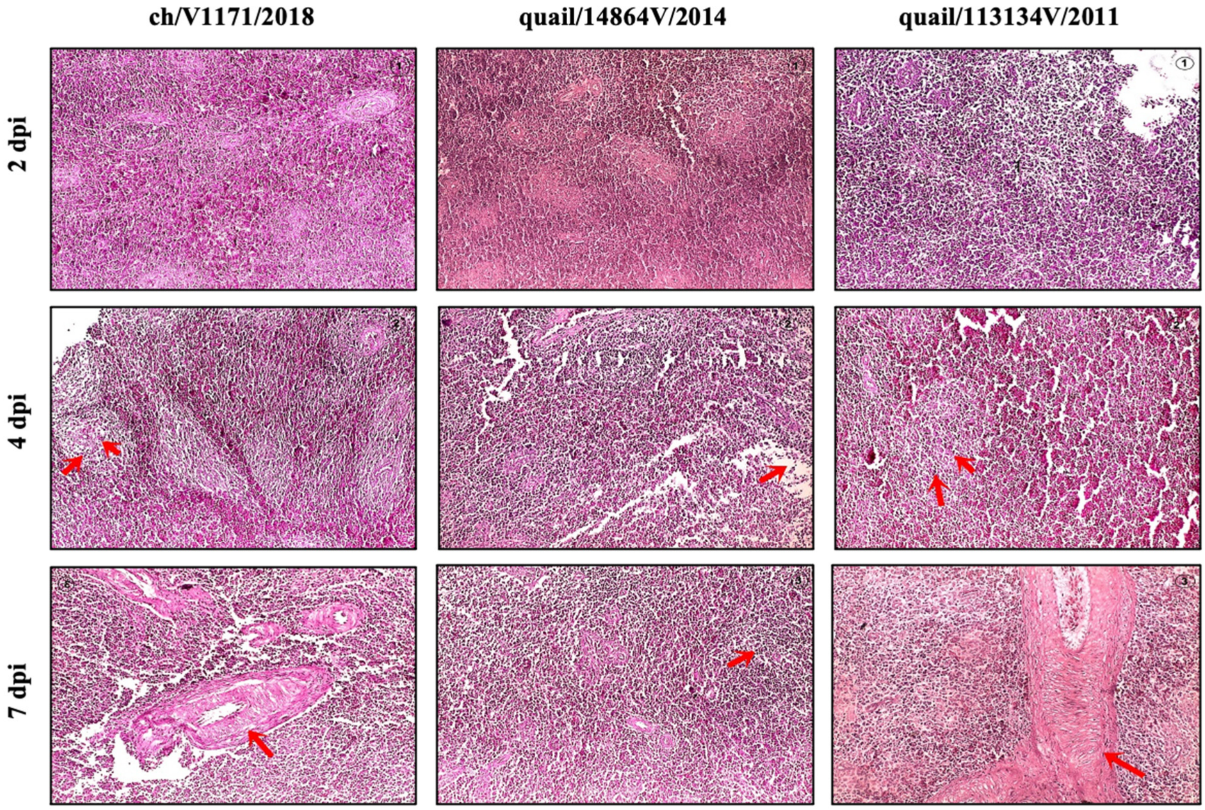

Supplementary Figure S7. Pathological changes of bursa.

Ch/V1171/2018: normal histological picture of the bursa (1), marked lymphocytic depletion (2), severe bursitis with thickening of the cortex (arrow) and proliferation of fibrous tissue within the medulla (3). **Quail/14864V/2014**: mild lymphocytic depletion (1), moderate lymphocytic depletion (2), marked depletion of the medulla with fine fibrous tissue proliferation (arrow) (3). **Quail/113134V/2011**: mild lymphocytic depletion (arrow) (1), bursitis with marked hemorrhage (red arrow) and lymphocytic depletion (arrow) (2), noticeable bursitis with cortical thickening and depletion of lymphoid follicles with fine fibrous tissue proliferation (3). **H&E (X 200)**

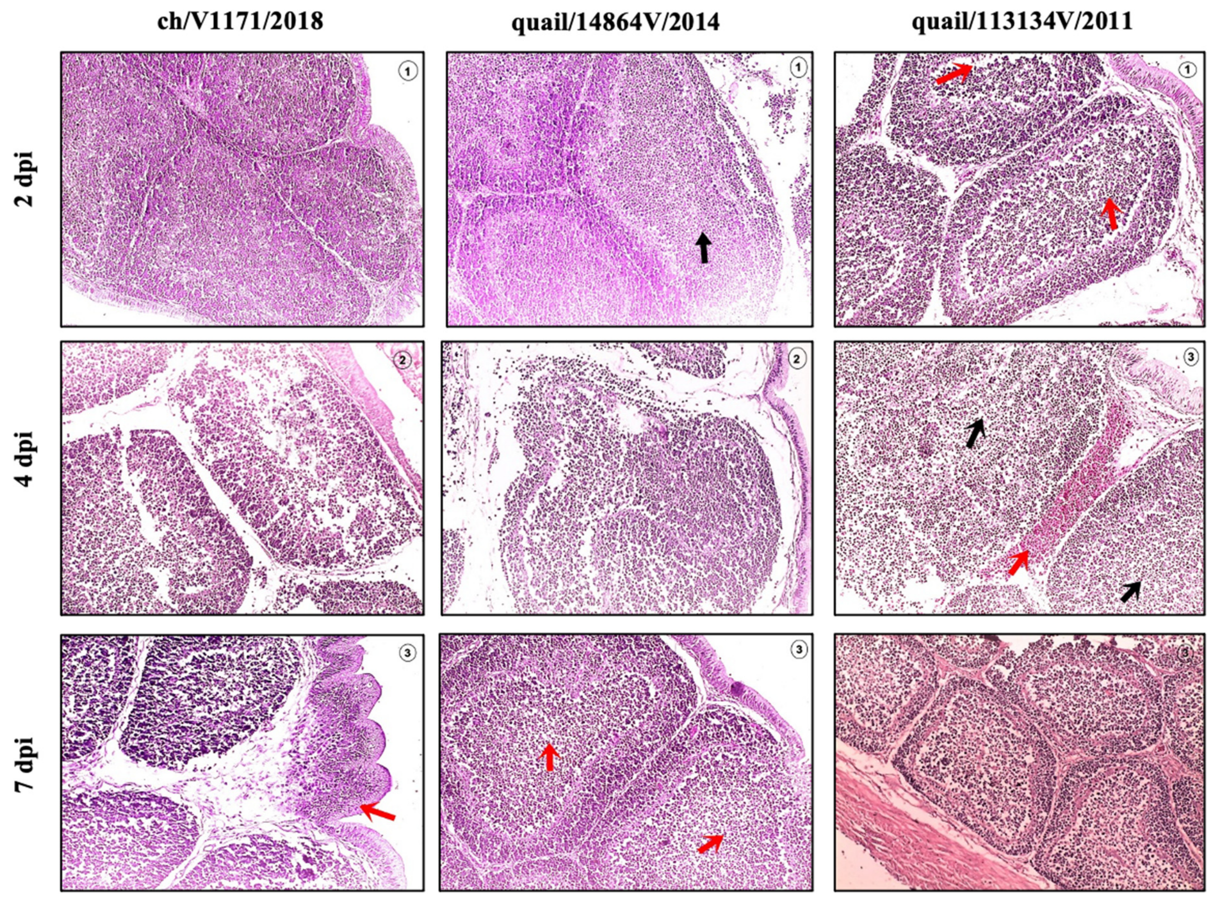

Supplementary Table S1. H9N2 viruses isolated and sequenced in this study

Virus ID	Location	Isolation date	Accession number
A/chicken/Egypt/17CAD74/2017	Beheira	2017-05-08	EPI1941118-25
A/chicken/Egypt/18V584/2018	Qalyubia	2018-02-25	EPI1941339-46
A/chicken/Egypt/18V1172/2018	Monufia	2018-03-01	EPI1941347-54
A/chicken/Egypt/18V1410/2018	Asyut	2018-04-28	EPI1941355-63

Supplementary Table S2. Histopathological findings for 3groups of chickens infected: Ch/V1171/2018 (Group1); Quail/14864V/2014 (Group 2); Quail/113134V/2011 (Group 3)

**Days**	**Group**	**Trachea**	**Lung**	**Intestine**	**Pancreas**	**Spleen**	**Bursa**	**Kidney**
**2 dpi**	**group1**	normal	- mild congestion of pulmonary<br>-blood vessels hemorrhages within the lung parenchyma	- mild enteritis	normal	normal	- normal	interstitial hemorrhages
	**group2**	marked activation of mucous glands	-mild pneumonia<br>-lymphocytic cells infiltration inside pulmonary blood vessels	-moderate enteritis<br>-vacuolation of epithelial lining and necrosis	normal	normal	mild lymphocytic depletion	interstitial hemorrhages
	**group3**	-marked activation of mucous glands<br>-submucosal edema	-congestion of blood vessels<br>-mononuclear inflammatory cells	moderate enteritis with vacuolation of epithelial lining and necrosis	mild edema	mild lymphocytic depletion	mild lymphocytic depletion	-interstitial hemorrhages<br>- tubular degeneration
**4 dpi**	**group1**	-moderate tracheitis<br>-submucosal edema and hemorrhages -lymphocytic cells infiltrations in lamina propria	-mild pneumonia with lymphocytic cells infiltrations<br>-proliferation of fibrous tissue within the parenchyma	-severe enteritis<br>-mononuclear cells infiltration<br>-vacuolation of the epithelial lining<br>-hyperplasia of intestinal glands	- pancreatitis<br>- vasculitis and lymphocytic cells infiltration	mild lymphocytic depletion	marked lymphocytic depletion	-mild tubular degeneration<br>-lymphocytic cells infiltration
	**group2**	-severe tracheitis<br>-necrosis of lining epithelium<br>-submucosal edema	-severe pneumonia<br>-marked hemorrhages and vasculitis with thrombus formation	-severe enteritis<br>-mononuclear cells infiltration<br>-vacuolation of the epithelial lining<br>-hyperplasia of intestinal glands and thickening of the muscular layer	pancreatitis	mild lymphocytic depletion	lymphocytic depletion	mild tubular degeneration
	**group3**	-severe tracheitis<br>-hemorrhages<br>-lymphocytic cells infiltration	marked hemorrhages in the parenchyma and 3ry bronchi	-severe enteritis<br>-mononuclear cells infiltration and vacuolation of the epithelial lining<br>-hyperplasia of intestinal glands<br>-thickening of the muscular layer	-pancreatic necrosis<br>-marked lymphocytic cells infiltration	splenitis	marked lymphocytic depletion	nephritis with peritubular lymphocytic cells infiltration

**7 dpi**	**group1**	-epithelial lining cells appeared to be necrosed<br>-lymphocytic cells infiltrations	severe pneumonia with hemorrhages inside the alveoli and vasculitis	-severe enteritis<br>-degeneration of lining epithelial<br>-severe lymphocytic cells infiltration.	- severe pancreatitis with interstitial edema and necrosed pancreatic acinar	-severe splenitis<br>-vasculitis	- severe bursitis with thickening of the cortex<br>-proliferation of interstitial fibrous tissue	severe nephritis
	**group2**	-Marked mononuclear cells infiltration<br>-hemorrhages within lining epithelium.	-fibrinous pneumonia<br>-noticeable lymphocytic cells infiltration within the lung parenchyma	-severe enteritis<br>-degeneration of lining epithelial<br>-severe lymphocytic cells infiltration	-Vasculitis<br>-lymphocytic cells infiltration	mild lymphocytic depletion	-severe bursitis<br>-thickening of the cortex<br>-proliferation of interstitial fibrous tissue	severe nephritis with lymphocytic cells infiltration
	**group3**	-Focal hyperplasia of the lining epithelium<br>-subepithelial lymphocytic infiltration	Marked vasculitis and pneumonia	-severe enteritis<br>-degeneration of lining epithelial<br>-severe lymphocytic cells infiltration	-pancreatic necrosis<br>-marked lymphocytic cells infiltration	splenitis	severe bursitis with thickening of the cortex and hemorrhages.	-severe nephritis<br>- lymphocytic cells infiltration

Supplementary Table S3. Quantitative Scoring of histopathological changes in examined organs (data are shown as mean ± SD)

**Organ**	**2dpi**			**4dpi**			**7dpi**		
**Trachea**	**Group 1**	**Group 2**	**Group 3**	**Group 1**	**Group 2**	**Group 3**	**Group 1**	**Group 2**	**Group 3**
congestion	0.2 ± 0.45^{a}	1.8 ± 0.45^{a}	1.2 ± 0.45^{a}	1.2 ± 0.45	1.4 ± 0.55	1.8 ± 0.45	3 ± 0.0^{c}	2.2 ± 0.45^{c}	2.6 ± 0.55
submucosal edema	0.2 ± 0.45^{a}	1.4 ± 0.55^{a}	2.8 ± 0.45^{a}	1.6 ± 0.55^{b}	2.2 ± 0.45	2.4 ± 0.55^{b}	2.6 ± 0.55	2.8 ± 0.45	2.8 ± 0.45
Mucosal necrosis	0.0 ± 0.0	0.2 ± 0.45	0.4 ± 0.55	0.6 ± 0.9^{b}	2.2 ± 0.45^{b}	0.2 ± 0.45	2.4 ± 0.55^{c}	1.2 ± 0.45^{c*}	2.6 ± 0.55*
Activation of mucous gland	0.0 ± 0.0^{a}	0.6 ± 0.55^{a}	3 ± 0.0^{a}	0.2 ± 0.45^{b}	0.6 ± 0.55*	1.6 ± 0.55^{b*}	0.0 ± 0.0^{c}	2.8 ± 0.45^{c}	3 ± 0.0^{c}
**Lung**									
Congestion/hemorrhages	2 ± 0.7	2.4 ± 0.55	2 ± 0.0	1.8 ± 0.45^{b}	2.2 ± 0.45*	3 ± 0.0^{b*}	2.6 ± 0.55	2.8 ± 0.45	3 ± 0.0
Inflammatory cells infiltration	1.6 ± 0.55	2.2 ± 0.45	1.6 ± 0.55	1.6 ± 0.55	1.6 ± 0.55	2.4 ± 0.55	2.6 ± 0.55	2.4 ± 0.55	2.4 ± 0.55
Necrosis of 3ry bronchi	1.2 ± 0.84	1.8 ± 0.45^{a}	0.4 ± 0.55^{a}	2 ± 0.0	2 ± 0.7	1 ± 0.7	2.8 ± 0.45	2.4 ± 0.90	2.4 ± 0.55
**Intestine**									
Inflammatory cells infiltration	0.8 ± 0.45	1 .3 ± 0.45	1.2 ± 0.45	1.2 ± 0.45	1 ± 0.0	1.4 ± 0.55	1.6 ± 0.55^{c}	2.4 ± 0.55^{c}	2.6 ± 0.55
Necrosis of lining epithelium	1.2 ± 0.45	1.4 ± 0.55	0.8 ± 0.45	1 ± 0.0	1.8 ± 0.45	1.4 ± 0.55	1.8 ± 0.45^{c}	2.8 ± 0.45^{c}	2.2 ± 0.45
**Pancreas**									
Edema	0.4 ± 0.55^{a*}	1.4 ± 0.55^{a}	1.2 ± 0.45*	0.4 ± 0.55^{b}	1.6 ± 0.55^{b}	1.6 ± 0.55^{b}	1.2 ± 0.45^{c}	2.2 ± 0.45^{c*}	1.2 ± 0.45*
Inflammatory cells infiltration	0.2 ± 0.45^{a}	1.2 ± 0.45^{a}	0.6 ± 0.55	0.6 ± 0.55^{b*}	1.4 ± 0.55*	1.4 ± 0.55^{b}	1.4 ± 0.55	1.8 ± 0.45	1.2 ± 0.45
Acinar necrosis	0.0 ± 0.0^{a}	1.2 ± 0.45^{a*}	0.4 ± 0.55*	0.2 ± 0.45^{b*}	1.6 ± 0.55*	1.4 ± 0.55^{b}	1.6 ± 0.55^{c}	2.4 ± 0.55^{c*}	1.4 ± 0.55*
**Spleen**									
Lymphocytic depletion	0.2 ± 0.45^{a}	0.6 ± 0.55*	1.4 ± 0.55^{a*}	1.6 ± 0.55	1.6 ± 0.55	0.2 ± 0.45	2.2 ± 0.45	1.6 ± 0.55	2.2 ± 0.45
Lymphocytic necrosis	0.0 ± 0.0^{a}	0.0 ± 0.0*	1.2 ± 0.45^{a*}	0.4 ± 0.55^{b}	0.2 ± 0.45*	1.6 ± 0.55^{b*}	1.2 ± 0.45^{c}	1.2 ± 0.45*	2.4 ± 0.55^{c*}
Fibrosis	0.0 ± 0.0^{a}	0.0 ± 0.0*	0.8 ± 0.45^{a*}	0.0 ± 0.0	0.0 ± 0.0	0.2 ± 0.45	1.2 ± 0.45^{c}	0.2 ± 0.45^{c}	2.2 ± 0.84^{c}
**Bursa**									
Lymphocytic depletion	0.2 ± 0.45^{a*}	1.2 ± 0.45^{a}	1.4 ± 0.55*	2.8 ± 0.45	2.2 ± 0.45	2.4 ± 0.55	2.2 ± 0.45	2.2 ± 0.45	2.6 ± 0.55
Lymphocytic necrosis	0.0 ± 0.0^{a*}	1 ± 0.7	1.6 ± 0.55*	2.2 ± 0.45^{b*}	1.2 ± 0.45^{b}	1.2 ± 0.45*	0.6 ± 0.55^{c}	1.6 ± 0.55^{c*}	2.8 ± 0.45*

Fibrosis	0.0 ± 0.0	0.0 ± 0.0	0.0 ± 0.0	0.4 ± 0.55	0.4 ± 0.55	0.2 ± 0.45	0.0 ± 0.0	0.4 ± 0.55	0.8 ± 0.84
**Kidneys**									
Congestion/hemorrhages	1.8 ± 0.45	1.8 ± 0.45	1.4 ± 0.55	1.4 ± 0.55 ^{b}	2.8 ± 0.45 ^{b}	2.2 ± 0.45 ^{b}	2.4 ± 0.55	2.4 ± 0.55	2.4 ± 0.55
Tubular degeneration	0.4 ± 0.55 ^{a}	1.8 ± 0.45 ^{a}	1.6 ± 0.55 ^{a}	2 ± 0.7	1.4 ± 0.55	2.2 ± 0.45	2.4 ± 0.55	2.6 ± 0.55	2.2 ± 0.45
Inflammatory cells infiltration	0.4 ± 0.55	0.4 ± 0.55	0.4 ± 0.55	1.8 ± 0.45	1.4 ± 0.55	1.4 ± 0.55	2.6 ± 0.55	2.6 ± 0.55	2.8 ± 0.45

Group1= Ch/V1171/2018; Group2 = Quail/14864V/2014; Group3 = Quail/113134V/2011

There are significant differences between groups with same superscript small litter/ asterisk at same row At *P*-value ≤ 0.05 using one-wayANOVA. ^{a} indicates the significance between the groups at 2dpi. ^{b} indicates the significance between the groups at 4 dpi.

^{c} indicates the significance between the groups at 7 dpi. * indicates the significance between the groups.

Supplementary Table S4. Antigenic characterization of G1 lineage H9N2 viruses

Antigen	Polyclonal antisera								
	v1172	v1410	CAD74	v3413	V864	FAO/FL5	Ir-10VIR-2008	Ch-KSA-VIR08	Mallard/Italy/3817-34/05
v1172-ch-2018	5120	5120	1576	1040	160	5120	5120	1280	2560
v1410 –ch-2018	2560	5120	4159	520	226	10240	10240	2560	5120
CAD74 –ch-2017	320	3880	3152	8317	113	2560	5120	160	640
v3413-qu-2011	320	1280	2560	10240	80	7240	10240	160	640
V864-qu-2014	122	50	320	25	905	40	40	80	160
FAO/FL5-ch-2015	243	1940	3152	10240	56	10240	10240	320	2560
Ir-10VIR-2008-G1/B	49	80	320	0	20	40	640	20	80
Ch-KSA-VIR08-G1/A	80	40	130	0	0	0	0	640	0
Mallard/Italy/3817-34/05	80	32	788	25	56	80	320	40	1280
